# N-Azacytidine Inhibits Myeloma Cell Growth While Preserving Multiple Myeloma Patient Derived-Bone Marrow Mesenchymal Stromal Cells Differentiation

**DOI:** 10.3390/metabo16080540

**Published:** 2026-07-31

**Authors:** Tamara Kukolj, Anica Spasić, Dragana Aleksandrović, Nikola Bogosavljević, Marko Vujačić, Mila Purić, Aleksandra Jauković, Drenka Trivanović

**Affiliations:** 1 Group for Hematology and Stem Cells, Institute for Medical Research, National Institute of the Republic of Serbia, University of Belgrade, 11000 Belgrade, Serbia; anicaspasic08@gmail.com (A.S.); dragana.aleksandrovic@imi.bg.ac.rs (D.A.); aleksandra@imi.bg.ac.rs (A.J.); 2 Center for Solid State Physics and New Materials, Institute of Physics, University of Belgrade, 11000 Belgrade, Serbia; 3 Institute for Orthopaedics Banjica, 11000 Belgrade, Serbia; nikola.bogosavljevic@iohbb.edu.rs (N.B.); marko.vujacic@iohbb.edu.rs (M.V.); 4 Clinic for Medical Oncology, Institute for Oncology and Radiology of Serbia, 11000 Belgrade, Serbia; mila.puric@ncrc.ac.rs

**Keywords:** multiple myeloma, N-azacytidine, epigenetic modulation, bone marrow mesenchymal stromal cells

## Abstract

Background/Objectives: Multiple myeloma (MM) is an incurable, hematological malignancy caused by the abnormal proliferation of terminally differentiated plasma cells in the bone marrow. Current research indicates that N-azacytidine, as an epigenetic drug and nucleoside metabolic inhibitor, can be efficient in MM treatment. However, data on bone marrow mesenchymal stromal cells (BMSCs), as a critical cell population in the hematopoiesis and bone remodeling processes, are limited. Methods: The effect of N-azacytidine on the myeloma cell line AMO-1, bone marrow mesenchymal stromal cells from MM patients (MM-MSCs) and patients undergoing hip arthroplasty (BMSCs) as a healthy control in hypoxia (3% O_2_) were examined. Cellular functions such as viability, proliferation, phenotype, and differentiation were determined by in vitro tests and flow-cytometry. Results: N-azacytidine (100 nM to 1000 nM) significantly inhibited metabolic activity, cell cycle, CFSE dilution and CD138+/CD38+ expression levels by myeloma cells after 72 h. No viability changes were detected in MM-MSCs and BMSCs treated with N-azacytidine. As for both BMSCs and MM-MSCs differentiation potential, N-azacytidine did not affect alkaline phosphatase (ALP) activity and adipogenesis but inhibited matrix mineralization (500 nM and 1000 nM). However, when BMSCs were pretreated with N-azacytidine for 72 h, changes in ALP activity and mineralization level were not detected. Conclusions: N-azacytidine, along with the suppressive effect on myeloma cells, did not change the differentiation potential of MSCs, but rather preserved their capacity. These findings are of particular importance considering MM progression and bone tissue destruction, suggesting that N-azacytidine may represent a promising therapeutic agent for myeloma disease.

## 1. Introduction

Multiple myeloma (MM) is a malignant hematological disorder that occurs due to uncontrolled proliferation of monoclonal plasma cells (terminally differentiated B-lymphocytes) in the bone marrow, disrupting the process of hematopoiesis and bone tissue remodeling [1,2]. According to the epidemiological data, MM accounts for 10% of all hematological malignancies, while the worldwide incidence of MM is currently 188,000, and mortality is 121,000 patients for the year 2022 [3].

MM usually develops gradually, through precursor stages: monoclonal gammopathy of undetermined significance (MGUS) and smoldering multiple myeloma (SMM) [4]. The diagnosis of final MM is established based on the presence of more than 10% of monoclonal plasma cells in the bone marrow, as well as the presence of one or more CRAB (calcium, renal failure, anemia, bone lesions) symptoms: elevated level of calcium (hypercalcemia), kidney failure, anemia, and bone lesions. Thus, dysregulated hematopoiesis and osteolytic bone lesions are key complications in MM [5,6].

At the cellular level, MM is the result of a series of sequential genetic and epigenetic changes that transform plasma cells, leading to the excessive production of monoclonal immunoglobulins or free light chains (M-protein). In addition to becoming the dominant cell population, transformed MM cells in bone marrow actively participate in bone tissue remodeling [4,7]. They stimulate the activity of osteoclasts via increased RANKL expression, while inhibiting osteoblasts that build bone matrix and the production of bone tissue components [8,9]. The result is the weakening of bone tissue and the development of osteolytic lesions, which are clinically manifested by bone pain, pathological fractures and reduced mobility [10,11].

Despite advances in the MM treatment [12,13], this malignancy remains an incurable disease, as almost all patients develop relapses [13]. Due to MM complexity, therapy requires a comprehensive approach to combine malignancy control but also accompanying symptoms, such as bisphosphonates for bone lesions and erythropoietic agents for anemia, to maintain patient quality of life [14,15]. Significant breakthroughs in the treatment of hematological malignancies have been achieved by the application of epigenetic therapeutics, which modulate reversible changes in gene expression [16].

Along with the common application in the treatment of myelodysplastic syndrome and myeloid leukemia [17], current research indicates that N-azacytidine, an epigenetic DNA methyltransferase inhibitor and metabolic reconfigurator, can also be beneficial in the MM treatment [18,19] as dysregulated DNA methylation and histone modification drive disease progression [20]. In addition to causing direct cytotoxicity of myeloma cells [18,21,22,23], N-azacitidine reprograms the bone marrow microenvironment in hematologic malignancies [24,25,26]. A special component of the bone marrow are mesenchymal stromal cells (BMSCs), a critical cell population in the hematopoiesis process, and at the same time representing the cellular basis for the bone tissue maintenance and remodeling [27,28,29]. Thus, since 95% of MM cells remain localized within the bone marrow, the stromal regulation is considered an indispensable therapeutic target in MM [30,31,32].

However, data addressing the influence of N-azacitidine on BMSCs of MM patients (MM-MSCs) is limited [33,34,35]. Here we demonstrate that in vitro administration of N-azacytidine has an inhibitory effect on the survival of AMO-1 myeloma cells, while preserving the osteogenic and adipogenic differentiation potential of BMSCs, derived from both MM patients and non-malignant controls. In this way, this work provides evidence that the use of N-azacitidine in MM patients at the appropriate dose can represent a promising therapeutic treatment with a protective role on cellular components of the bone marrow microenvironment.

## 2. Materials and Methods

### 2.1. Isolation and Cell Culture of MSC and AMO-1 Cells

Primary mesenchymal stromal cells were isolated from bone marrow of non-malignant patients (BMSCs) and multiple myeloma patients (MM-MSCs) after obtaining informed consent from the patients. The study was conducted in accordance with the Declaration of Helsinki. Bone marrow samples of non-malignant patients undergoing hip arthroplasty and bone marrow specimens from newly diagnosed MM patients were obtained from the Banjica Institute of Orthopedics, Belgrade, and the Institute of Oncology and Radiology of Serbia in Belgrade. Upon receipt in the lab, bone marrow samples were washed in PBS (Phosphate-Buffered Saline, Capricorn Scientific, Ebsdorfergrund, Germany) by centrifugation at 1400 rpm for 5 min. The obtained cell pellet was resuspended in PBS and filtered through nylon strainers with a pore size of 70 µm and centrifuged again at 1400 rpm for 5 min. Next, mononuclear cells were isolated based on density gradient centrifugation using Lymphoprep™ (Capricorn, Scientific, Ebsdorfergrund, Germany). Mononuclear cells were seeded in flasks (Thermo Fisher Scientific, Nunc, Waltham, MA, USA), and adherent BMSCs and MM-MSCs were expanded by cultivation in standard growth medium (GM) containing Minimal Essential Medium (MEM; Capricorn Scientific, Ebsdorfergrund, Germany) with 10% fetal bovine serum (FBS; Capricorn Scientific, Ebsdorfergrund, Germany), 1% penicillin/streptomycin (Capricorn Scientific, Ebsdorfergrund, Germany), 10 mM HEPES buffer (PanBiotech, Aidenbach, Germany) and 5 ng/mL fibroblast growth factor (FGF, BioLegend, San Diego, CA, USA). Cells were maintained at 37 °C in a humidified atmosphere containing 5% CO_2_ and a hypoxic chamber with 3% O_2_ (BioSpherix, Parish, NY, USA) with regular medium exchange. Upon reaching confluency, cells were detached from culture flasks by trypsin/EDTA (Capricorn, Scientific, Ebsdorfergrund, Germany), centrifuged, and washed, while the cell number was determined by Trypan-blue dye (Gibco, Grand Island, New York, NY, USA). All experiments were performed using MSC between passages 2 and 5.

A commercially available human cell line, multiple myeloma AMO-1, was obtained thanks to Prof. Dr. Regina Ebert, University of Würzburg, Germany. AMO-1 cell line was cultured in RPMI medium (Capricorn, Scientific, Ebsdorfergrund, Germany) supplemented with 10% FBS, 2 mM L-glutamine, and 1% penicillin/streptomycin (Capricorn, Scientific, Ebsdorfergrund, Germany) under hypoxic conditions (3% O_2_). Cells were seeded at an initial density of 1 × 10^5^ cells/mL and passaged upon reaching confluency, with medium exchange every 2–3 days.

### 2.2. Cellular Viability and Metabolic Activity: MTT Assay

Cell viability of both primary stromal cells and AMO-1 cells was assessed using the MTT test. AMO-1 cells were seeded in 96-well plates at a density of 2 × 10^4^ cells/well in 200 μL RPMI medium supplemented with 10% FBS and treated with N-azacytidine at concentrations of 50 nM, 100 nM, 200 nM, 500 nM, and 1000 nM for 24, 48, and 72 h under hypoxic conditions (3% O_2_). Following the treatment, 20 μL of MTT solution (3-(4,5-dimethiltiazol-2yl-)-2,5 diphenyltetrazolium bromide, Sigma Aldrich, Burlington, MA, USA) at a final concentration of 5 mg/mL was added to each well. After 3 h of incubation at 37 °C, the reaction was stopped with 10% SDS in 1 N HCl, dissolving formazan crystals. For adherent cultures, cells were seeded in 96-well plates at 5 × 10^3^ cells/well and allowed to attach for 24 h before treatment. After incubation with N-azacytidine for 24, 48, and 72 h, MTT solution (final concentration 5 mg/mL; Sigma-Aldrich, Burlington, MA, USA) was added, incubated for 3 h at 37°, while formazan crystals were dissolved in 100 μL isopropanol (VWR Chemicals, Rosny-sous-Bois, France). The optical density of the obtained solutions was measured at 540 nm using a microplate reader (Labsystems Multiskan PLUS, Helsinki, Finland).

### 2.3. Flow Cytometry Analyses of Surface Markers, Proliferation, and Cell Cycle

Expression of CD138 and CD38 surface markers on AMO-1 cells was analyzed by flow cytometry following 72 h of treatment with N-azacytidine (100 nM, 500 nM, and 1000 nM). Cells were collected, washed in cold PBS with 0.5% bovine serum albumin (Capricorn Scientific, Scientific, Ebsdorfergrund, Germany), and incubated for 45 min at 4 °C in the dark with fluorochrome-conjugated antibodies: anti-CD138-APC (BioLegend, San Diego, CA, USA) and anti-CD38-FITC (Beckman Coulter, Villepinte, France). Afterwards, cells were washed in PBS, fixed in 4% paraformaldehyde, and analyzed using a BD FACSLyric flow cytometer (BD Biosciences, San Jose, CA, USA). After 72 h treatment with N-azacytidine (100 nM, 500 nM and 1000 nM), the proliferative capacity of AMO-1 was assessed by Trypan blue exclusion assay, CFSE staining, and cell cycle analysis. Cell number was determined using Trypan blue dye to discriminate between live and dead cells. For cell division distribution, cells were labeled with 5 µM carboxyfluorescein succinimidyl ester (CFSE; Affymetrix, eBioscience, San Diego, CA, USA) in PBS supplemented with 5% fetal bovine serum (FBS; Capricorn Scientific, Ebsdorfergrund, Germany) for 15 min at room temperature in the dark. After washing, cells were seeded in 24-well plates at a density of 5 × 10^4^ cells/well and treated with N-azacytidine (Sigma-Aldrich, St. Louis, MO, USA) for 72 h under hypoxic conditions (3% O_2_). The distribution of the dividing AMO-1 cell population was determined based on the CFSE dilution analyzed by flow cytometry. For cell cycle analyses, following the treatment, cells were collected, washed in cold PBS, fixed in 4% paraformaldehyde, and stained with 1 ug/mL DAPI (4′,6-diamidino-2-phenylindole) (Sigma-Aldrich). DNA content was analyzed by flow cytometry. Flow cytometry was performed using a Cytomics FC 500 cytometer (Cytomics FC 500, Beckman Coulter, Brea, CA, USA). Data was processed using FlowJo v10 software (Tree Star, Ashland, Ashland, OR, USA).

### 2.4. Immunofluorescence Staining and Microscopy

For Immunofluorescence analysis, BMSCs and MM-MSCs were seeded on glass coverslips in 24-well plates (2 × 10^3^ cells/coverslip) and cultivated without or with N-azacytidine (100 nM, 500 nM, and 1000 nM) for 72 h under hypoxic conditions (3% O_2_). Cells were fixed, permeabilized with 0.1% Triton X-100 (Sigma Aldrich, Burlington, MA, USA), and blocked in PBS containing 1% BSA for 30 min at room temperature. Then, primary antibodies were added: mouse anti-NANOG, rabbit anti-OCT-4, and mouse anti-SOX-2 (all from Cell Signaling Technology, Danvers, MA, USA). Phalloidin–tetramethylrhodamine B isothiocyanate (Sigma-Aldrich) at a concentration of 10 µg/mL was used to stain F-actin. Following the primary antibody incubation, samples were washed 3 times with PBS, and the corresponding FITC-coupled secondary antibodies (Sigma-Aldrich) were added for 2 h. Nuclei were counterstained with 1 ng/mL DAPI (Sigma-Aldrich). Next, cells were washed, and coverslips were mounted with Fluoromount™ Aqueous Mounting Medium (Sigma Aldrich, Burlington, MA, USA). Samples were analyzed using an epifluorescence microscope (Olympus, Tokyo, Japan). Signal quantification was performed by image densitometry in NIH—ImageJ software version 1.54k (LOCI, University of Wisconsin, Madison, WI, USA). For densitometric analysis, images of cells in wells were assessed, with the same image size and magnification used.

### 2.5. Osteogenic and Adipogenic Differentiation Potential of MSCs

The differentiation potential of BMSCs and MM-MSCs towards osteogenic and adipogenic lineages was examined in vitro under hypoxic conditions. Cells were seeded in 96-well plates at a concentration of 5000 cells/well and, upon reaching subconfluency, GM was replaced with the osteogenic induction medium (OM) or adipogenic induction medium (AM) without or with N-azacytidine (100 nM, 500 nM, and 1000 nM). For OM, GM without FGF was used and contained dexamethasone (10 nM; Applichem GmbH, Darmstadt, Germany), 50 μM ascorbic acid (Sigma-Aldrich), and 10 mM β-glycerophosphate (Sigma-Aldrich, Burlington, MA, USA). As for the N-azacytidine pretreatment effect, MSC were cultivated in GM without or with N-azacytidine (100 nM, 500 nm, and 1000 nM) for 3 days, and subsequently only in OM for an appropriate time. Early osteogenic differentiation was assessed after 7 days by analyzing alkaline phosphatase (ALP) activity by staining with BCIP/NBT solution (Sigma-Aldrich, Burlington, MA, USA), while the late phase of differentiation, i.e., matrix mineralization, was assessed after 14 days by Alizarin red staining (Riedel de Haen, Hanover, Germany). A light microscope (Olympus, Tokyo, Japan) was used to capture cells, while the osteogenic degree was quantified by image densitometry in NIH—ImageJ software version 1.54k (LOCI, University of Wisconsin, Madison, WI, USA). For densitometric analysis, images of cells in wells were assessed, with the same image size and magnification used. Adipogenic differentiation was induced by GM (without FGF) containing 1 μM dexamethasone, 100 μg/mL isobutyl-methylxanthine (IBMX; Sigma-Aldrich), and 10 μg/mL insulin (Sigma-Aldrich). After 14 days of cultivation, lipid droplets were detected by Oil Red O staining (Merck, Darmstadt, Germany), and the level of differentiation was assessed microscopically (Olympus, Tokyo, Japan), by counting Oil Red O-positive cells.

### 2.6. Colony-Forming Unit-Fibroblast Test (CFU-F)

To assess the clonogenicity of BMSCs and MM-MSCs, a Colony-Forming Unit-Fibroblast (CFU-F) assay was performed. Cells were seeded in 24-well plates (at a concentration of 100 cells/well) in the presence of N-azacytidine (100 nM, 500 nM, or 1000 nM) and cultivated in hypoxic conditions for 10 days in GM or OM. Next, cells were fixed in cold methanol and stained with Crystal violet dye. The percentage of formed colonies relative to the total number of seeded cells in each well was determined as colony efficiency.

### 2.7. Statistical Analysis

Statistical analysis was performed using GraphPad Prism 10 software (GraphPad Software, San Diego, CA, USA). The number of investigated patients’ specimens (biological replicates) is indicated as *n* for each experiment in the figure legends. Differences between groups were evaluated using the nonparametric Kruskal–Wallis and Two-Way ANOVA tests, followed by appropriate post hoc analysis. A value of *p* < 0.05 was considered statistically significant.

## 3. Results

### 3.1. N-Azacytidine Inhibits Viability, Proliferative Capacity and Modulates the CD138/CD38 Myeloma Phenotype of the AMO-1 Cell Line

**Figure 1:**
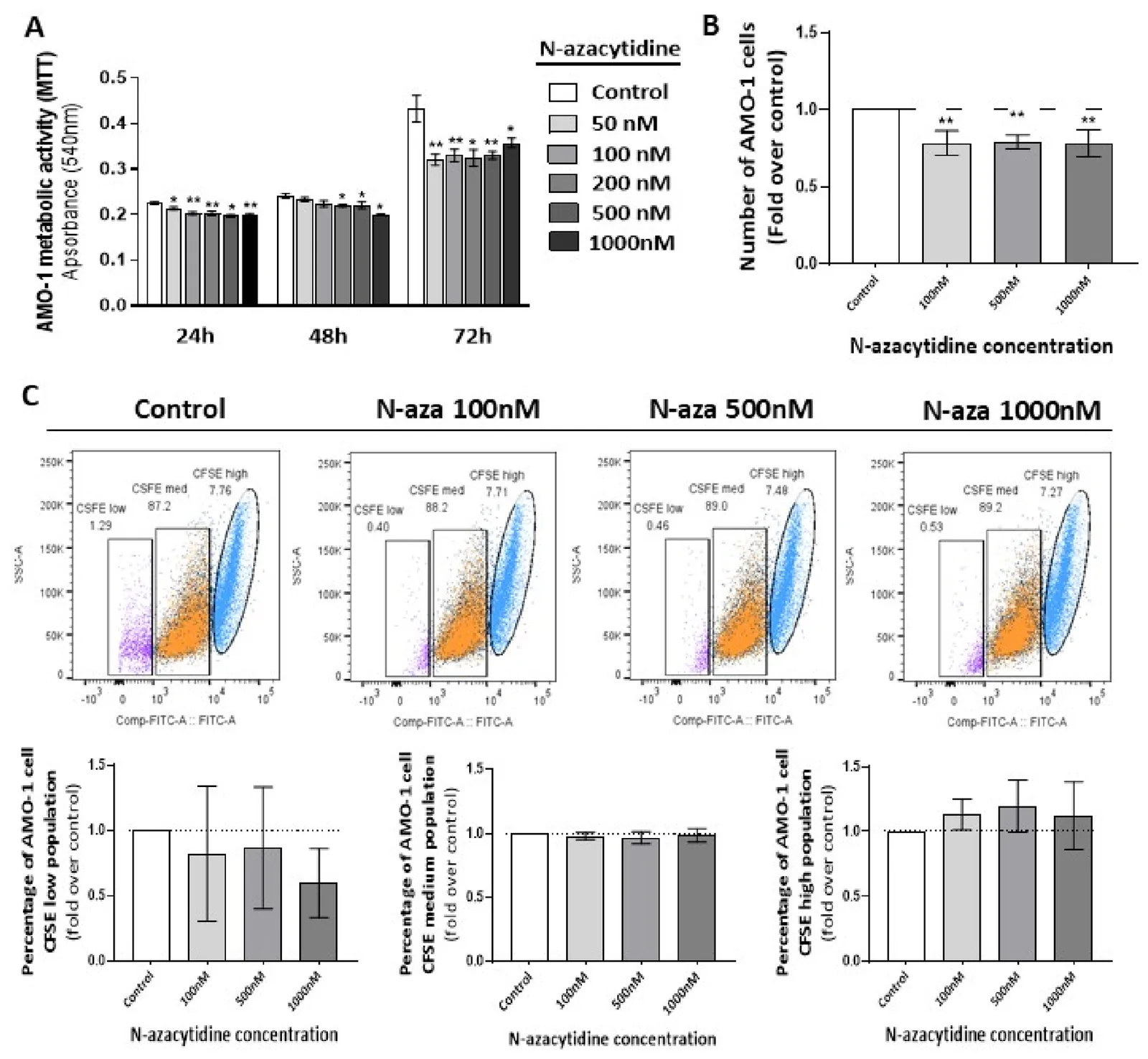

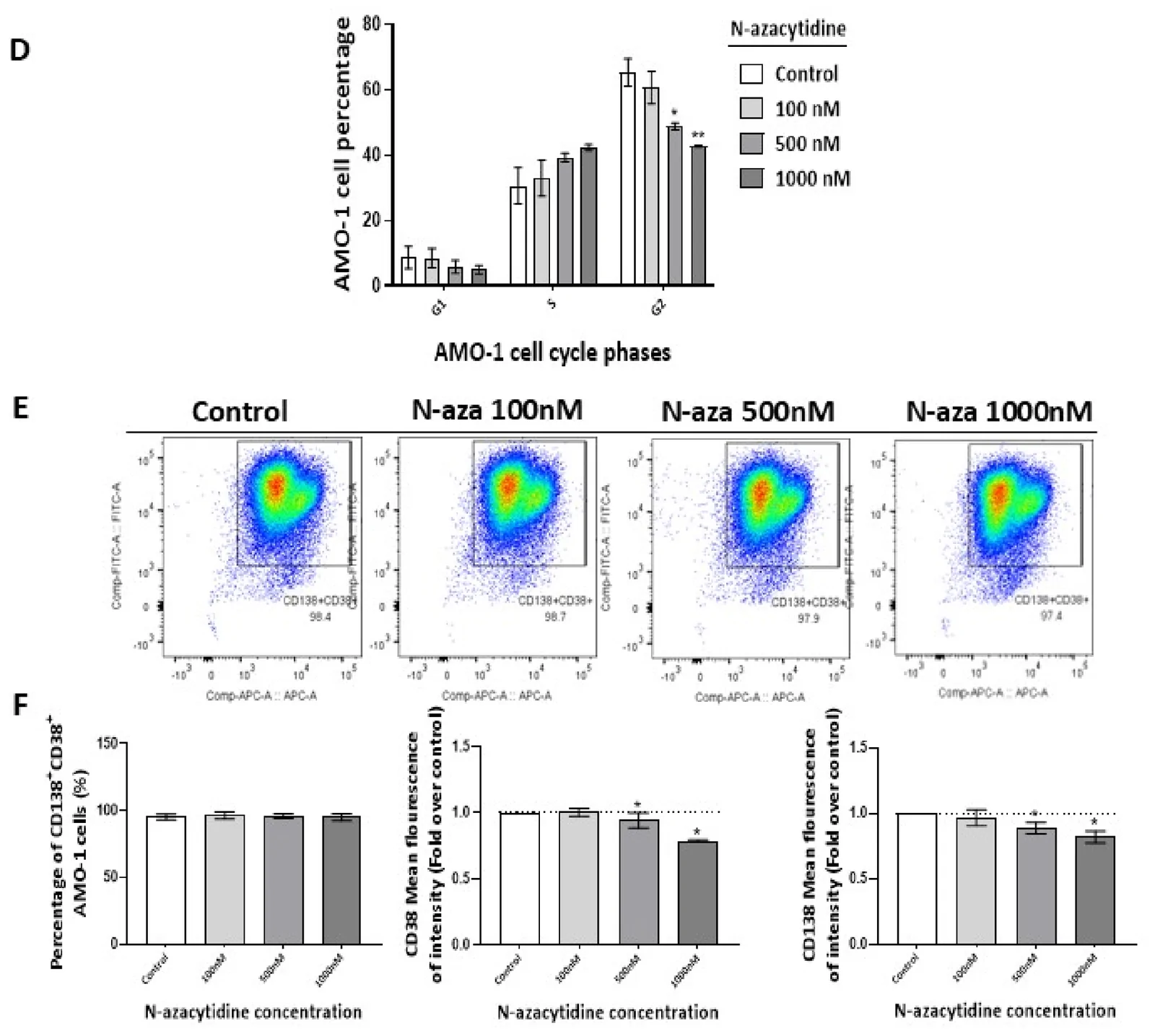
The effect of N-azacytidine (N-aza) on the viability and proliferative capacity of a myeloma cell line AMO-1 under hypoxic conditions (3% O_2_). (**A**) Metabolic activity of AMO-1 cells treated with N-aza (0, 50 nM, 100 nM, 200 nM, 500 nM, and 1000 nM) during 24 h, 48 h, and 72 h was determined using the MTT test. Results in graphs are presented as mean ± SEM from at least three independent experiments. (**B**) Number of viable AMO-1 cells after 72 h treatment with N-aza estimated by using Trypan blue solution. Graphical results are shown as mean ± SEM of at least three independent experiments and normalized to untreated control. (**C**) Proliferation rate of AMO-1 cells analyzed by CFSE staining after 72 h treatment with N-aza. Representative dot-plots of total CFSE-stained cell populations. Graphical display of cell populations percentages labeled as CFSE low, medium, and high. Results in graphs are presented as mean ± SEM from at least three independent experiments, performed in triplicate, and normalized to untreated control. (**D**) Cell cycle progression of AMO-1 cells determined by DAPI staining using flow cytometry. The percentages of cells distributed in the cell cycle phases are represented. Results are shown as mean ± SEM of at least three independent experiments and normalized to untreated control. (**E**) Representative dot-plots of CD138^+^, CD38^+^ AMO-1 cells. (**F**) Results for percentages of CD138^+^CD38^+^ coexpressing cells and mean fluorescence intensity of CD138^+^ and CD38^+^ positive cells. Results are shown as mean ± SEM of at least three independent experiments and normalized to untreated control. Statistical significance was determined using the Kruskal–Wallis test: ** *p* < 0.01; * *p* < 0.05 relative to the untreated control.

Viability of AMO-1 cells in the presence of increasing concentrations of N-azacytidine (50–1000 nM) at different time points (24 h, 48 h, and 72 h) was assessed by the MTT test. The results showed a statistically significant reduction in metabolic activity that correlates with the concentration and treatment time increase, whereby the greatest decline (the viability of AMO-1 cells about 80%) was observed after 72 h (Figure 1A). In line with this result, counting of AMO-1 cells treated with N-azacytidine (100 nM, 500 nM, and 1000 nM) during 72 h, demonstrated a significant reduction in cell proliferation rate of approximately 20%+/−4.49% across all groups (representing the maximum SEM among the groups) (Figure 1B). Cell division analyzed by CFSE labeling demonstrated separation of three cell populations in the analyzed AMO-1 cells treated with N-azacytidine (100 nM, 500 nM, and 1000 nM) after 72 h. Results demonstrated the reduction trend of the CFSE low population (actively dividing cells), as well as an increase in the CFSE high population (cells that have undergone few or no divisions) under the influence of N-azacytidine, implying the inhibitory effect of N-azacytidine on the proliferative capacity of AMO-1 cells (Figure 1C). Analyses of cell cycle progression of AMO-1 cells following the 72 h treatment with N-azacytidine (100 nM, 500 nM, and 1000 nM) showed a reduction in cell distribution in G2 phase (Figure 1D). Along with proliferative capacity, results revealed that N-azacytidine treatment had not changed the percentage of AMO-1 myeloma cells coexpressing CD138 and CD38 (approximately 98.7–97.4% of positive cells) (Figure 1E). However, further analysis of the fluorescence intensity of CD138^+^ and CD38^+^ positive cells showed that N-azacytidine at concentrations of 500 nM and 1000 nM significantly reduced the intensity of the fluorescence signal (Figure 1F), which indicates that N-azacytidine treatment may still be important for the modulation of the phenotype of myeloma cells, as well as the subsequent mechanisms controlled by these markers.

### 3.2. Comparison of Morphological Characteristics, Differentiation Potential, and Viability of MM-MSCs and BMSCs

**Figure 2:**
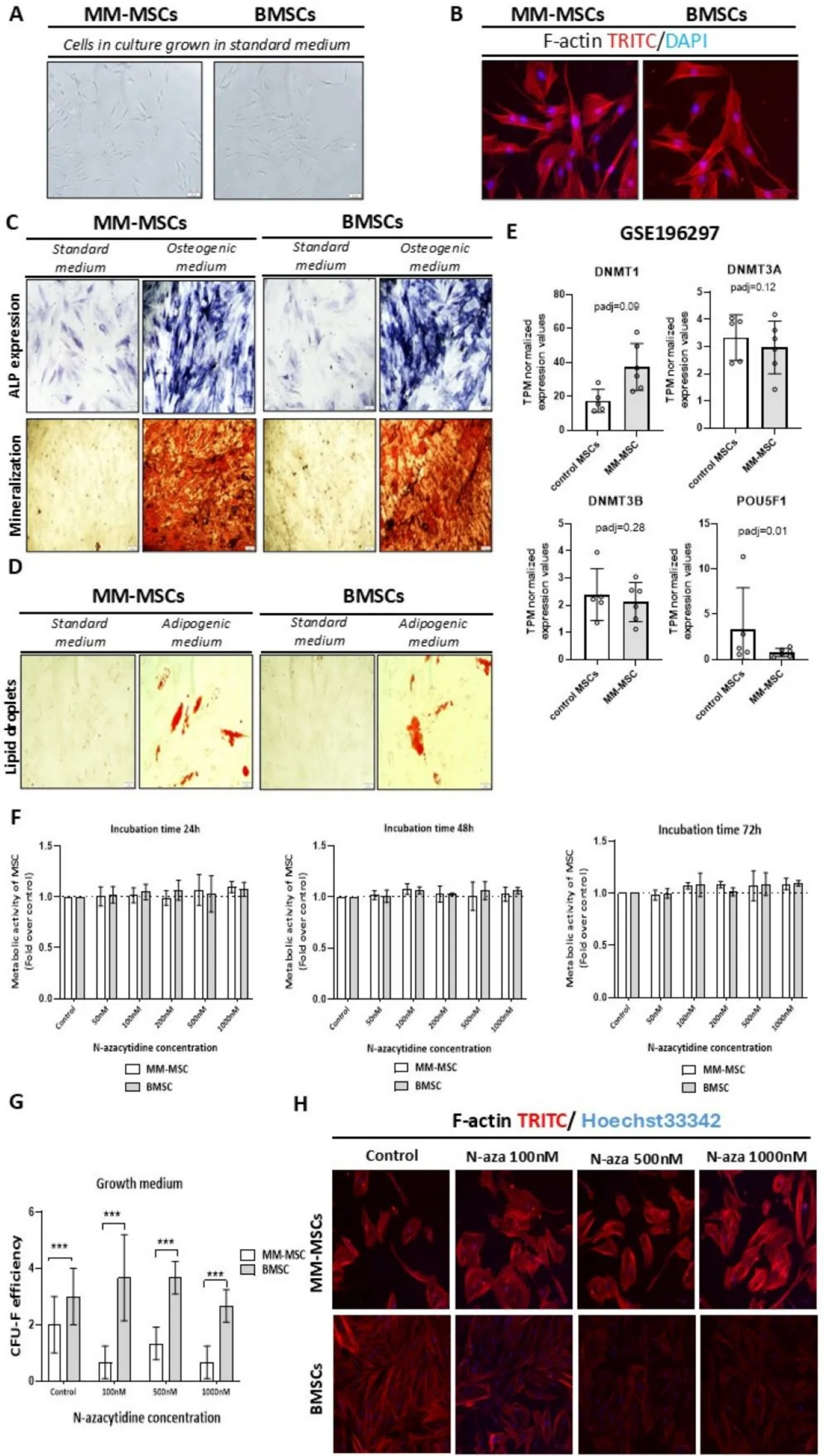
Cellular features of MM-MSCs and BMSCs: morphology, actin organization, differentiation potential, viability, and clonogenicity. (**A**) Adherent, fibroblast-like cells grown in hypoxia (3% O_2_), in standard medium for 3 days. Scale bar: 100 µM. (**B**) Representative micrographs of immunofluorescence microscopy of F-actin and nuclei detected by DAPI stain (blue). Scale bar: 50 μm. (**C**) Osteogenic differentiation assessed after 7 days of cultivation by staining for alkaline phosphatase (ALP) activity and after 14 days for mineralization by Alizarin red. Representative micrographs of cells are presented. Scale bar: 50 μm. (**D**) Intracellular lipid droplets obtained after adipogenic induction and Oil Red O after 14 days. Representative micrographs of cells are presented. Scale bar: 20 μm. (**E**) Analysis of differentially expressed genes from dataset GSE196297 in myeloma compared to normal bone marrow, including key genes involved in DNA methylation and pluripotency. TPM normalized expression values are shown for DNMT1, DNMT3A, DNMT3B, and POU5F1 (OCT4) are shown in control MSCs (*n* = 5) and MM-MSCs (*n* = 6). Results are presented as mean ± SD. (**F**) Influence of N-aza on the metabolic activity (viability) of MM-MSCs and BMSCs under hypoxic conditions (3% O_2_). Cells were seeded in 96-well cell culture plates (5000 cells/well) and treated with different concentrations of N-aza (0, 50 nM, 100 nM, 200 nM, 500 nM, and 1000 nM). Metabolic activity was determined after 24 h, 48 h, and 72 h using the MTT test. The results are shown as mean ± SD of at least three independent experiments, performed in triplicate, and normalized to control (untreated cells). The number of patient specimens *n* = 4. (**G**) Cellular clonogenicity in the presence of N-aza. Cells were seeded in 24-well plates (100 cells/well) and cultured in standard medium for 14 days with N-aza (0, 100 nM, 500 nM, and 1000 nM) under hypoxia (3% O_2_). The Colony-Forming Unit-Fibroblast (CFU-F) number of MM-MSCs and BMSCs are presented in the graph as means ± SD from at least three independent experiments. The number of patient specimens *n* = 4. Statistical significance was determined using Two-Way ANOVA test: *** *p* < 0.001 relative to the corresponding group between MM-MSCs and BMSCs. (**H**) Actin organization after 72 h of treatment with N-aza (0, 100 nM, 500 nM, and 1000 nM) in hypoxia (3% O_2_). Representative micrographs of immunofluorescence microscopy of F-actin and nuclei detected by DAPI (blue). Scale bar: 50 μm.

Next, we established mesenchymal stromal cell cultures derived from bone marrow of MM patients (MM-MSCs) and non-malignant control (BMSCs) to precisely determine the effect of N-azacytidine on MM-MSCs and compare them to BMSCs. To observe potential differences, we investigated their basic characteristics. Both cell types are fibroblast-like in shape, whereby this morphology was maintained even during long-term cultivation through passages (Figure 2A). In addition, expressions of cytoskeletal proteins, including F-actin (Figure 2B), show similarity in its organization in both cell populations.

Bearing in mind the importance of osteogenic and adipogenic differentiation in the bone marrow, a comparative analysis confirmed the ability of osteogenesis and adipogenesis of MM-MSCs and BMSCs, while no differences were observed in their differentiation potential (Figure 2C). The obtained results showed that both cell populations exhibited a significant increase in the expression of the enzyme alkaline phosphatase (ALP) when cultured in osteogenic medium compared to cells grown in standard medium (control), as an indicator of the early phase of osteogenic differentiation. In addition, after long-term incubation in the osteogenic medium, Alizarin red staining of deposited calcium into the extracellular matrix did not show a difference between MM-MSCs and BMSCs (Figure 2C). Moreover, based on Oil Red O staining, MM-MSCs and BMSCs can be differentiated into adipocytes with formed lipid droplets, with no difference between cells observed (Figure 2D). Although additional analysis (e.g., in vivo) is necessary, these basic tests indicated that there was no significant difference in the stem cell properties between MM-MSCs and BMSCs.

Differential gene expression analysis was performed using datasets GSE196297 [36] from the GEO database to compare MSCs from the control group (non-malignant bone marrow, control MSCs) and active MM disease (MM-MSCs). DNMT1 and POUF51 (OCT4) genes were upregulated, while DNMT3A and DNMT3B were slightly reduced in MM-MSCs, when compared to control MSCs (Figure 2E). This suggested hypomethylating agents, such as N-azacytidine, might be efficient in the regulation of proteins of importance for MM-MSCs integrity. The results of the MTT test demonstrate that N-azacytidine does not cause significant changes in the metabolic activity of stromal cells. It does not affect the viability of these cells at any of the investigated time points, as opposed to exhibiting an inhibitory effect on myeloma cells (Figure 2F). However, the efficiency of CFU-F formation showed significantly lower potential in MM-MSCs compared to BMSCs. Although not statistically significant, a different trend of the effect of N-azacytidine on MM-MSCs and BMSCs was observed. While the number of BMSCs colonies increased when treated with N-azacytidine, MM-MSCs showed decreased ability to form colonies in the presence of N-azacytidine (Figure 2G). Interestingly, in MM-MSCs, N-azacytidine increased, while in BMSCs, it reduced F-actin staining (Figure 2H). This suggests that the N-azacytidine may remodel the cytoskeleton in a context-dependent manner, leading to different responses in MM-MSCs and BMSCs to epigenetic modulation, which could influence cell shape, adhesion, migration, and interactions within the bone marrow microenvironment.

### 3.3. N-Azacytidine Modulates Pluripotency Markers Expression in MM-MSCs and BMSCs

**Figure 3:**
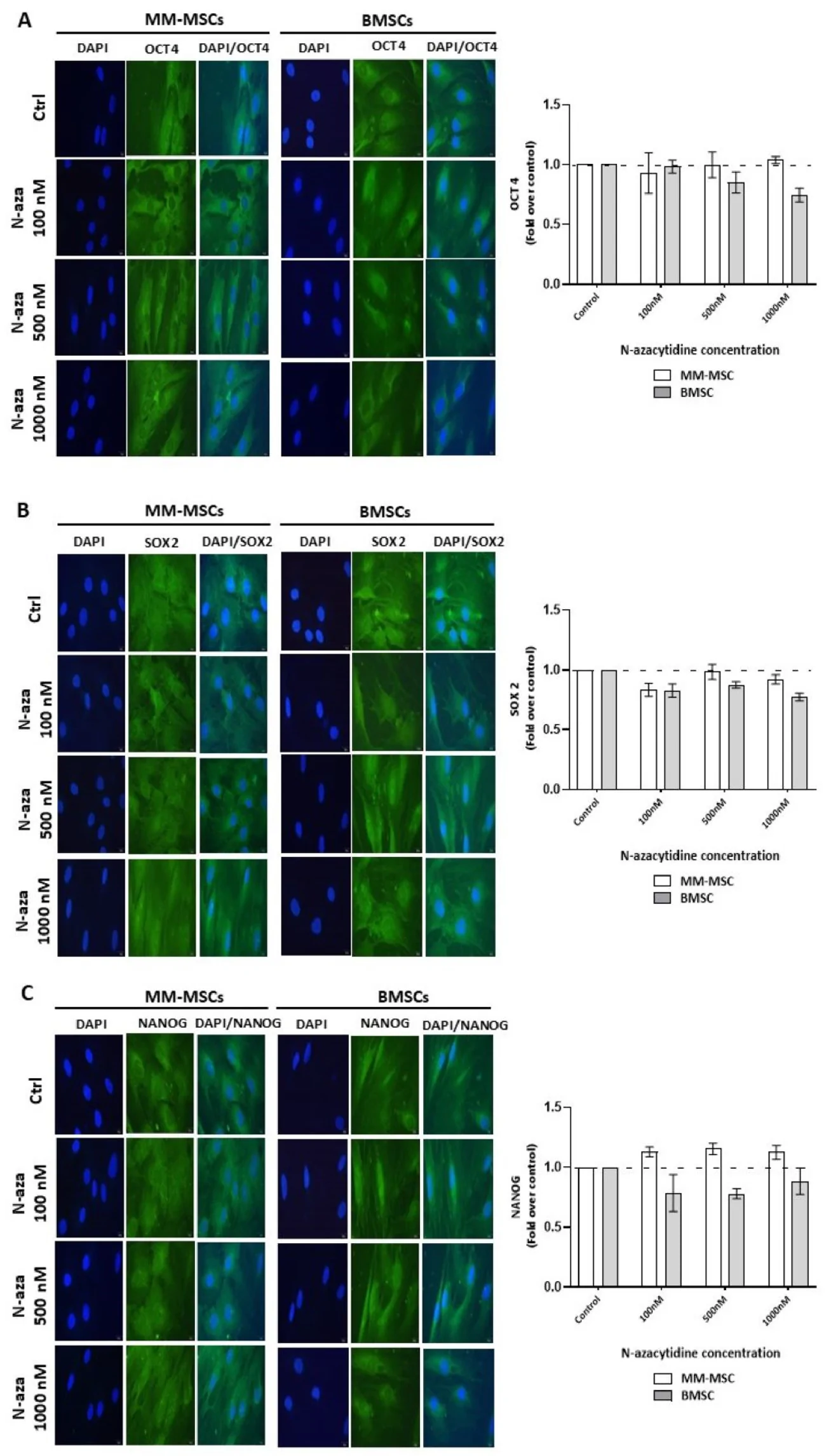
Expression of pluripotency-related transcription factors in MM-MSCs and BMSCs exposed to N-aza (0, 100 nM, 500 nM, and 1000 nM) during 72 h in low oxygen conditions (3% O_2_). Micrographs of: (**A**) OCT4; (**B**) SOX-2, and (**C**) NANOG detected by indirect immunofluorescence staining with FITC-conjugated corresponding secondary antibodies. Nuclear DNA was stained with DAPI (blue). Scale bars: 20 μm. Representative images of three independent experiments are shown. Signal quantification was performed by image densitometry in NIH—ImageJ software (LOCI, University of Wisconsin, Madison, WI, USA). Results in graphs are presented as means ± SD of four different patient specimens (*n* = 4) assessed in at least three independent experiments. Values are normalized to the control (cells in differentiation medium without N-aza).

At the molecular level MSC self-renewal capacity, stemness retention and clonogenic proliferation rates are directly determined by pluripotency marker expression, such as OCT4, NANOG, and SOX2. Figure 3A–C shows expressions of these markers in MM-MSCs and BMSCs determined by immunofluorescence labeling, following the N-azacytidine treatment. Both cell types showed positive expression of OCT4, SOX2, and NANOG. We observed a lack of baseline nucleus localization of pluripotency-related markers in MM-MSCs. Although not statistically significant, N-azacytidine decreased OCT4, SOX2 and NANOG protein expression in BMSCs that was followed by the reduced localization in the nucleus. On the contrary, this was not the case in MM-MSCs. Nevertheless, these results indicate that N-azacytidine has a different action potential on the expression of pluripotency markers depending on the MSC source. At the level of the MM bone marrow niche, it is important that MM-MSCs maintain the expression of these markers in the presence of N-azacytidine, i.e., stemness capacity.

### 3.4. Effect of N-Azacytidine on the Differentiation Potential of MM-MSCs and BMSCs: Osteogenesis and Adipogenesis

**Figure 4:**
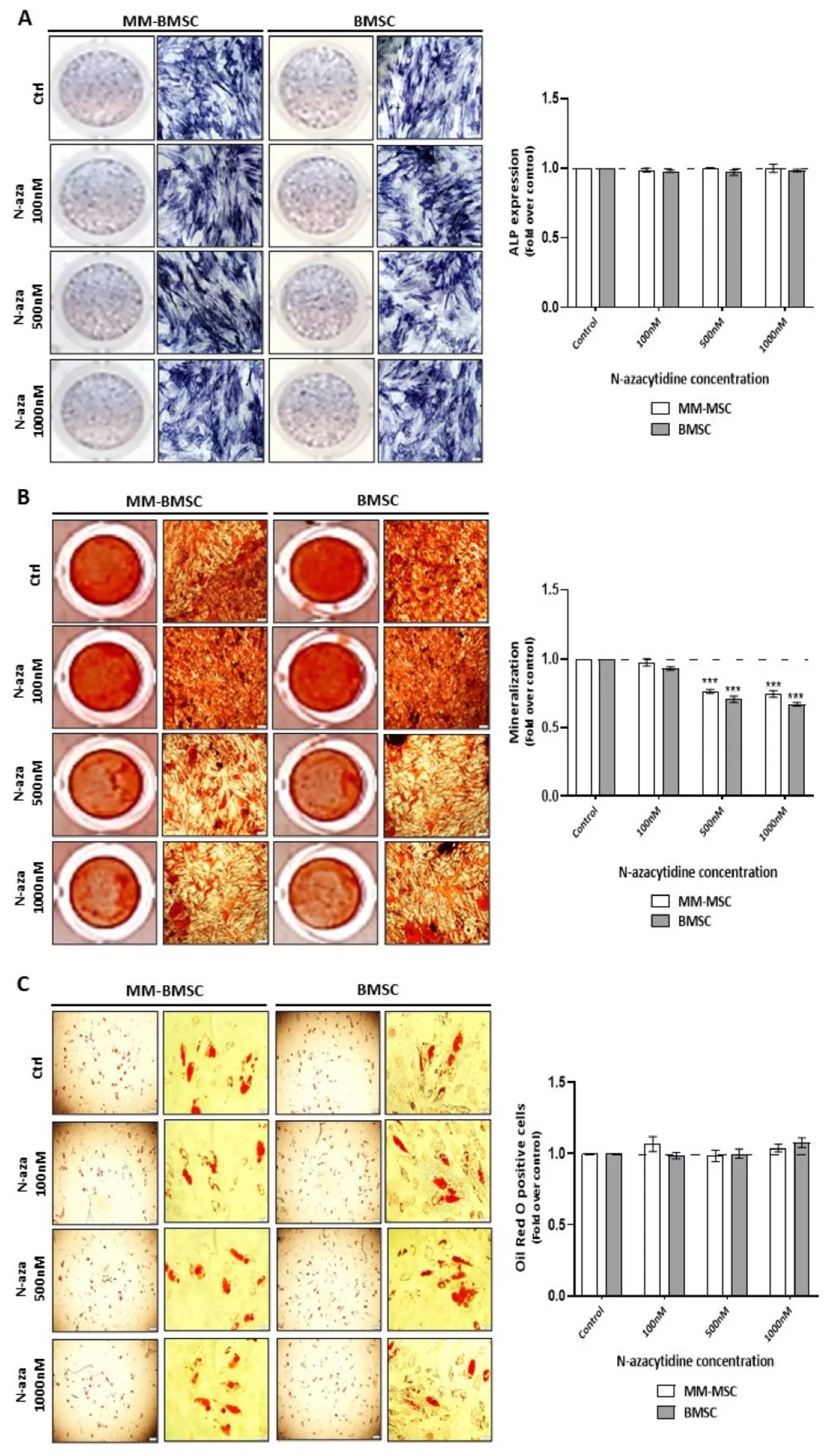
Osteogenic and adipogenic potential of MM-MSCs and BMSCs in the presence of N-aza. Cells were grown under hypoxic conditions (3% O_2_) in growth medium until confluency and then induced for differentiation in the appropriate medium in the presence of N-azacytidine (N-aza). (**A**) Alkaline phosphatase (ALP) activity detected after 7 days of treatment in osteogenic medium. Representative images of wells and cells are shown. Scale bar: 50 µm. (**B**) Extracellular matrix mineralization determined by Alizarin red staining after 14 days of treatment in osteogenic medium. Representative images of wells and cells are shown. Scale bar: 50 µm. (**C**) Adipogenic differentiation was assessed based on the number of cells with formed lipid droplets after 14 days in adipogenic medium. Representative images of wells and cells are shown. Scale bar: 10 µm and/or 50 µm. Results in graphs are presented as means ± SD of four different patient specimens (*n* = 4) assessed in at least three independent experiments. Values are normalized to the control (cells in differentiation medium without N-aza). Statistically significant difference in comparison with control in the absence of N-aza by Kruskal–Wallis test: *** *p* < 0.001.

By using alkaline phosphatase (ALP) enzyme detection assay, no significant differences in the presence of N-azacytidine were determined, both in MM-MSCs and BMSCs after 7-day culture in osteogenic medium (Figure 4A). This finding indicates that N-azacytidine did not alter the process of early phase osteogenic differentiation. In addition, Alizarin red was used to detect the ability of cells to form a mineralized matrix after 14 days of treatment in an osteogenic medium (Figure 4B). The obtained results show that N-azacytidine induces a similar response in MM-MSCs and BMSCs regarding the degree of mineralization. Namely, the concentration of N-azacytidine of 100 nM does not lead to significant changes in the mineralization level of the extracellular matrix compared to the corresponding untreated control in both MM-MSCs and BMSCs. However, in the presence of higher concentrations of this drug (500 nM and 1000 nM), a statistically significant decrease in mineralization compared to the control was shown (Figure 4B). Therefore, it can be concluded that N-azacytidine, although it does not affect the initial phase of osteogenic differentiation, has the potential to impair the formation of mineralized extracellular matrix in later stages of the bone-forming process. On the other hand, Oil Red O staining of lipid droplets in adipogenic-differentiated cultures demonstrated that N-azacytidine (100 nM, 500 nM, and 1000 nM) does not affect adipogenesis in both MM-MSCs and BMSCs. Namely, based on the number of cells in which lipid droplets were formed, no significant differences were found in the presence of N-azacytidine compared to untreated cells. Altogether, N-azacytidine selectively preserved early osteogenic commitment and adipogenic differentiation, but when applied at higher concentrations (≥500 nM), it impaired late-stage bone matrix mineralization. This suggests that at lower concentrations (100 nM), N-azacytidine maintained BMSCs’ osteogenic and adipogenic potential, which is important for preserving bone marrow function in MM treatment.

### 3.5. Effect of N-Azacytidine Pretreatment on the MM-MSC and BMSC Osteogenesis and Clonogenicity Under Osteogenic Conditions

**Figure 5:**
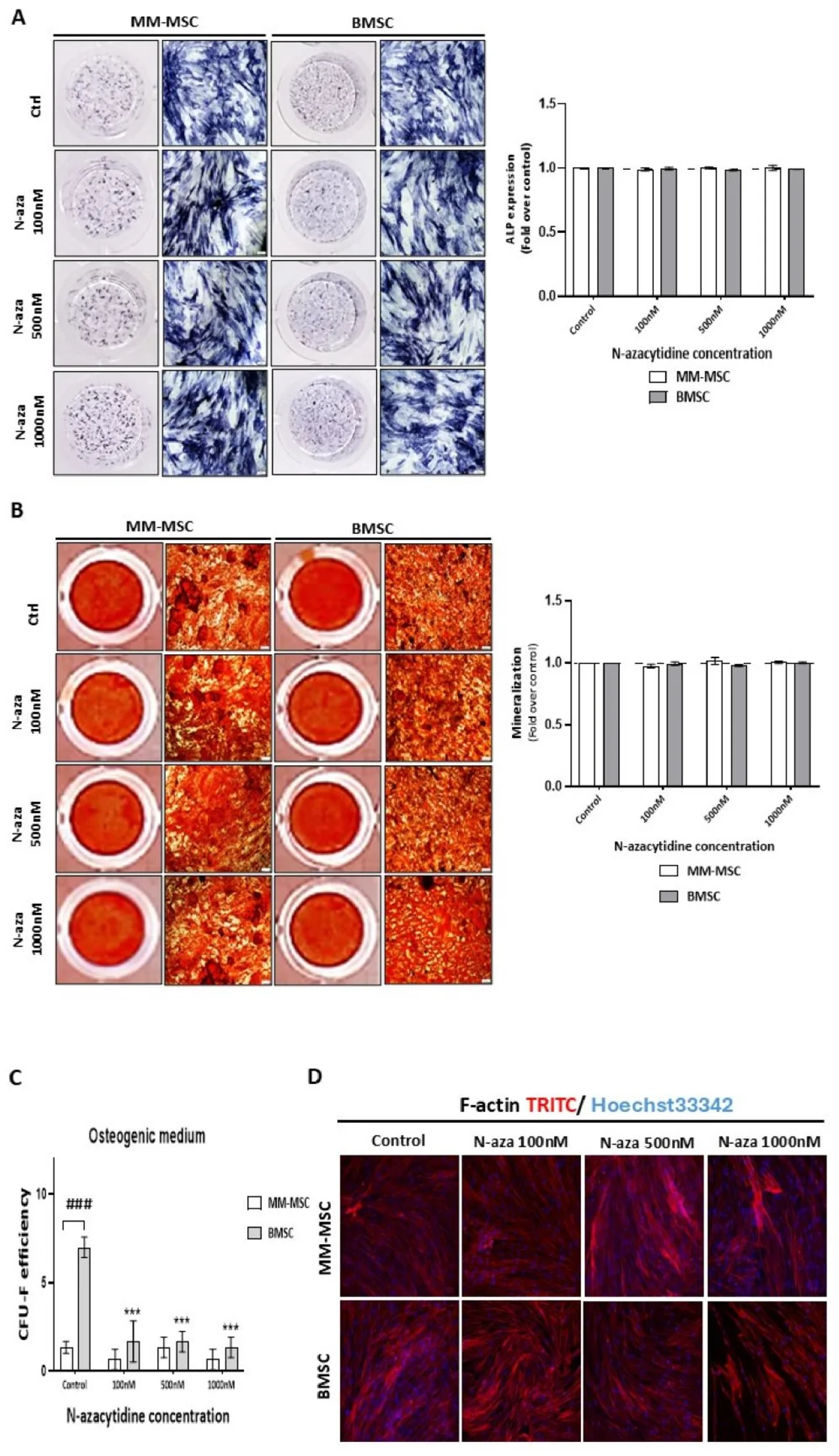
The effect of N-azacytidine (N-aza) pretreatment on osteogenic differentiation of MM-MSCs and BMSCs. Cells were grown under hypoxic conditions (3% O_2_) in a growth medium, treated with N-aza (0, 100 nM, 500 nM, and 1000 nM) for 72 h. Then the treatment was removed, and cells were induced to osteogenesis. (**A**) Alkaline phosphatase (ALP) expression was detected after 7 days in osteogenic medium. Representative images of wells and cells are shown. Scale bars: 50 µm. (**B**) Extracellular matrix mineralization determined by Alizarin red staining after 14 days of treatment in osteogenic medium. Representative images of wells and cells are shown. Scale bars: 50 µm. Values are normalized to the control (cells in differentiation medium without N-aza). Results in graphs are presented as means ± SD of four different patient specimens (*n* = 4) assessed in at least three independent experiments. (**C**) Cellular clonogenicity in osteogenic conditions in the presence of N-aza. Cells were seeded in 24-well plates (100 cells/well) and cultivated in osteogenic medium for 14 days with N-aza (0, 100 nM, 500 nM, and 1000 nM) under hypoxia (3% O_2_). The Colony-Forming Unit-Fibroblast (CFU-F) number of MM-MSCs and BMSCs results are presented in graphs as means ± SD from at least three independent experiments. Statistical significance was determined using Two-Way ANOVA test: *** *p* < 0.001 relative to the untreated control or; ### *p* < 0.001 in comparison between MM-MSC and BMSCs. (**D**) Actin organization after 72 h of treatment with N-aza in osteogenic medium under hypoxia (3% O_2_). Representative micrographs of immunofluorescence microscopy of F-actin and nuclei detected by DAPI (blue). Scale bar: 50 μm.

As the biggest changes were determined at the level of osteogenic differentiation, we further conducted additional experiments to fully elucidate the effect of N-azacytidine on the osteogenic potential of MM-MSC and BMSCs. Given that the real conditions of the therapeutic application of chemotherapeutics imply a certain period, after which the therapy is paused, in this study we tried to simulate those conditions in vitro. We investigated the effect of pretreatment of these cells with N-azacytidine, i.e., the capacity of MSC to establish the original capacity of osteogenic differentiation, following the treatment. Namely, cells were grown for 72 h in the presence of N-azacytidine (100 nM, 500 nM, and 1000 nM) in the standard medium, after which the medium was changed, and further induction continued only in the osteogenic medium for 7 or 14 days. The obtained results showed that this treatment regimen did not affect the expression of ALP enzyme, either in MM-MSC or in MM-MSC (Figure 5A). Next, results showed that this pretreatment regime also did not modify the ability of the mineralization process of the extracellular matrix, neither in MM-MSCs, nor in BMSCs (Figure 5B), which altogether indicates that the effect of N-azacytidine on osteogenesis is reversible, and that N-azacytidine did not lead to loss of osteogenic potential of MM-MSCs and BMSCs. In addition, the graph of colony efficiency in osteogenic conditions (Figure 5C) also shows that, as in the growth medium, BMSCs have a higher colony formation capacity compared to MM-MSCs. However, unlike the effects in the growth medium, N-azacytidine statistically significantly reduces the efficiency of BMSCs to form colonies but does not affect the ability of MM-MSCs to form colonies. Therefore, it can be concluded that in the osteogenic setting, N-azacytidine shows different reactivity of clonogenic efficiency depending on the type of cells, but that the colony formation potential is not lost, both in BMSCs and in MM-MSCs.

## 4. Discussion

In this study we aimed to investigate the potential of an epigenetic and metabolic modulator, N-azacytidine, to inhibit the growth of the myeloma cells, along with emphasis on bone marrow mesenchymal stromal cells (BMSCs) response. Our results show that this drug, in addition to stopping the proliferative capacity of malignant cells, simultaneously maintains the osteogenic potential of both control BMSCs and BMSCs derived from MM patients (MM-MSCs).

N-azacytidine, an epigenetic DNA methyltransferase inhibitor (DNMTi), has been shown to have multiple mechanisms of action in hematologic malignancies, both on cancer cells, as well as on the tumor microenvironment [37,38,39,40,41]. As recent encouraging findings indicate [18,19,42], N-azacytidine may be a significant candidate for further clinical trials and a potential addition to modern, clinical therapeutic protocols for MM treatment. However, the effect of this drug in MM patients is still unclear, requiring more detailed investigations related to the dosage, interaction response with other MM drugs, and long-term impact on the myeloma bone marrow niche.

In this study we used AMO-1 myeloma cell line as a well-established line from a relapsed MM patient with a representative phenotype, being an excellent model for exploring anti-myeloma drugs [43]. Our results clearly confirm that N-azacytidine (50–1000 nM) effectively inhibits the growth of AMO-1 myeloma cells. After 72 h of treatment, AMO-1 cells showed a significant reduction in metabolic activity, proliferation rate, and cell cycle progression of myeloma cells. These findings are supported by several studies showing that this drug also works across different myeloma cell lines, while the effect is dependent on the dose and time of treatment [21,44,45,46]. Importantly, even a ~20% reduction in MM cell viability is biologically significant in the context of epigenetic therapy, which often works via differentiation or sensitization rather than direct cytotoxicity. Analysis of CD138 and CD38 expression, two most important phenotypic markers for identification and monitoring plasma cells in MM [46,47], showed that N-azacytidine does not affect the total percent of CD138+CD38+ AMO-1. However, deeper analysis revealed reduced expression of these molecules on individual cells in the presence of N-azacytidine, suggesting its potential to alter phenotypic characteristics linked to malignant cell survival and MM progression [48,49]. Although qualitative changes were not observed, small to moderate changes in the surface density of these proteins directly alter cell signaling, metabolic rates, adhesion dynamics, and microenvironmental interactions. Fluctuations in marker density directly modulate how strongly a cell senses microenvironmental signals, adheres to matrix structures, and processes metabolic substrates [50,51,52,53]. By continuously degrading NAD^+^, CD38 directly controls intracellular and extracellular NAD^+^/NADH ratios, altering glycolysis and mitochondrial function promoting tumor survival [54,55,56]. Thus, inhibited CD38 expression by N-azacytidine could be the underlying mechanism that directly reverses the metabolic state of MM cells, triggering cell cycle arrest and tumor cell death, as detected in our study. Along with CD38, CD138 binds and concentrates essential growth factors (like IL-6, HGF, and VEGF) at the cell surface that support tumor growth [57]. Therefore, inhibition of CD138 caused by the action of N-azacytidine may also be an additional mechanism involved in the reduction in metabolic activity, cell cycle progression and proliferation of myeloma cells. Namely, CD38 is the direct target for highly successful monoclonal antibodies like Daratumumab and Isatuximab. The quantitative density of CD38 on the cell surface directly dictates the efficacy of these therapies. Lower expression or shedding of CD138 correlates with advanced disease stages, high-risk features, and poor overall survival. Nevertheless, due to the complex role of CD38 and CD138 in MM and their significance in therapy [50,51,52,53,58,59,60,61]–further analysis is needed to elucidate the contribution of N-azacytidine in regulating the expression of these key markers for tracking myeloma cells.

Since BMSCs are an important contributor in bone marrow niche regulation, their status is crucial, but in MM it may be altered [62,63,64,65]. Research by [31] revealed that DNMT inhibitors in MM revert pathological MSC state to a healthy state, restoring their osteogenic potential and suppressing pro-tumor inflammatory cytokines like IL-6 and TNF-α. Comparison of basic morphology and differentiation potential between BMSCs and MM-MSCs in our study showed no differences, whereby the overall sensitivity to N-azacytidine was largely similar between BMSC and MM-MSCs, as well as their reactivity to N-azacytidine (50–1000 nM). Unchanged stromal cell viability in the presence of higher doses of N-azacytidine (50–1000 nM) is consistent with the published data [44,66,67]. Previous studies reported high resistance of BMSCs to N-azacytidine depending on the dose and treatment duration [66,67,68,69,70] whereby it is suggested that the effects on BMSCs proliferation and cell cycle can be reversible [68]. In our study, the most significant differences between BMSCs and MM-MSCs were determined at the level of colony formation capacity based on the CFU-F test. BMSCs showed a significantly higher efficiency. Although additional analyzes are needed, this result may be explained as the consequence of their functional abnormalities developed due to residence in the tumor microenvironment [71]. In addition, differences in colony formation potential and F-actin organization in the presence of N-azacytidine were also detected. Although the result is not statistically significant, N-azacytidine stimulated colony formation in BMSCs and inhibited the intensity of F-actin staining. In contrast, in MM-MSCs, N-azacytidine led to a decrease in the number of colonies formed, but more intense F-actin staining. Bearing in mind the importance of F-actin organization for numerous cellular functions, including the maintenance of stem cell pull [72], it can be speculated that there is a correlation between colony formation and cytoskeleton organization in the presence of F-actin in MSCs from the bone marrow. In brief, increased colony formation of BMSCs in the presence of N-azacytidine, along with decreased F-actin level may imply improved stemness capacity, in contrast to the trend achieved by MM-MSC. However, immunofluorescence analysis also showed that N-azacytidine may decrease pluripotency marker expression (OCT4, NANOG, and SOX2) in BMSCs, with no changes induced in MM-MSC, indicating different responsiveness of these cells on this drug. Moreover, we considered F-actin and pluripotency markers might be involved in the N-azacitidine effects shown on MSC clonogenicity and differentiation (main stem cell properties). Namely, the F-actin cytoskeleton acts as a dynamic biophysical processor that links stemness, clonogenicity, and cell fate/differentiation [68]. Since different responses in clonogenicity, actin organization, and pluripotency marker expression were detected after treatment in MM-MSCs and BMSCs, it may be speculated that cellular source, i.e., the specific microenvironment that MM-MSC and BMSC come from, underlie these differences. Overall, it can be concluded that additional analyses are necessary to elucidate the molecular mechanisms underlying the effect of N-azacytidine on the stemness properties and fate of bone marrow MSCs.

N-azacytidine was also reported to induce various changes in differentiation potential of stromal cells [73,74,75,76]. In the context of bone marrow niche and MM, we were focused on investigating MSCs osteogenesis and adipogenesis. Our study shows that N-azacytidine (100–1000 nM) does not significantly affect the early phase of osteogenic differentiation, but in higher concentrations (500 and 1000 nM) leads to reduced extracellular mineralization matrix, while no differences were observed in the response between MM-MSCs and BMSCs. On the contrary, data from [68,76,77] imply pro-osteogenic potential of N-azacytidine. As for adipogenesis, it has been reported that BMSCs from MDS and AML patients show increased adipogenic potential [78,79]; however, we did not detect differences in terms of BMSCs and MM-MSCs potential, i.e., MM. Also, no changes in presence of N-azacytidine were noticed, although its stimulatory influence on lipid droplets formation at 1 uM and 10 uM was published [77,80]. These discrepancies with previous data may be due to different cell sources, inter-individual donor variations, and cultivation conditions. Still, considering that N-azacytidine had the most pronounced effect on the ability to mineralize the extracellular matrix of MSCs, we further extended analysis of osteogenic potential. We analyzed the effect of pretreatment to determine the degree of reversibility of osteogenic potential after treatment, capacity of colony formation in osteogenic medium and actin organization. Following the 72 h of treatment, N-azacytidine was removed, cells were induced to osteogenesis and no changes in osteogenic potential in both BMSCs and MM-MSCs were detected. The sustained expression of ALP, as well as the reversible capacity of mineralization, indicate that under these conditions the differentiation potential of MSC may remain preserved. Moreover, in BMSCs, N-azacytidine reduced colony efficiency under osteogenic settings, while this was not the case in MM-MSCs, implying again different reactivity of these two cell types in terms of colony formation in presence of N-azacytidine. In the osteogenic medium, there are also differences in the organization of F-actin, both between BMSCs and MM-MSCs and in their response to N-azacytidine. As the increased polymerization of actin is related with promoted osteogenesis [81], we may assume that in MM-MSCs N-azacytidine in higher doses (500 nM and 1000 nM) could support osteogenic commitment, unlike in BMSCs. Overall, these results indicate that the effect of N-azacytidine on osteogenesis is reversible, i.e., N-azacytidine does not impair the osteogenic potential of bone marrow MSCs, while additional molecular analyses are needed to reveal mechanisms of its action.

Nevertheless, this study has several limitations that should be acknowledged. First, although we used a single myeloma cell line (AMO-1), which limits generalizability across MM, the selected AMO-1 cell line is a well-established line from a relapsed MM patient with a representative phenotype, being an excellent model for exploring anti-myeloma drugs [43]. Next, the small biological sample size (limited patient numbers) for both BMSCs and MM-MSCs may not fully capture inter-patient variability. Further studies, with additional patients need to be performed, including samples from treatment-naïve and relapsed patients, to better characterize donor-to-donor variability and to assess niche interactions in more physiological contexts (xenografts). However, directly comparing non-malignant versus MM-derived MSCs addresses a critical question on how the N-azacytidine affects the bone marrow niche in disease versus non-cancer conditions. Although our in vitro study lacks in vivo or ex vivo validation to confirm whether observed effects translate to the disease context, obtained results justify progression to in vivo models and combination therapy studies in future work.

## 5. Conclusions

Here, we showed the in vitro effects of the DNMT inhibitor N-azacytidine as a potential therapeutic agent for myeloma. Since N-azacytidine is already approved for myelodysplastic syndromes (MDS) and acute myeloid leukemia (AML), demonstrating its efficacy in myeloma while preserving bone marrow mesenchymal stromal cell function has promising translational potential. This study addresses a critical gap by showing anti-myeloma activity without compromising the osteogenic niche—an important advance given the myeloma bone. Along with decreased metabolic activity, proliferation, cell cycle progression, and CD138 and CD38 expression in myeloma cells, here we demonstrated that N-azacytidine does not interfere with osteogenesis and adipogenesis of stromal cells of both multiple myeloma (MM-MSCs) and non-malignant patients (BMSCs). Although additional analysis is needed, these results indicate that N-azacytidine may be considered as a significant additional or adjuvant drug used in the therapy of MM.

## Data Availability

All data generated during this study are included in this published article.
